# Optimal inequalities for bounding Toader mean by arithmetic and quadratic means

**DOI:** 10.1186/s13660-017-1300-8

**Published:** 2017-01-25

**Authors:** Tie-Hong Zhao, Yu-Ming Chu, Wen Zhang

**Affiliations:** 1 School of Mathematics and Computation Sciences, Hunan City University, Yiyang, 413000 China; 2 Icahn School of Medicine at Mount Sinai, Friedman Brain Institute, New York, 10033 United States

**Keywords:** 26E60, arithmetic mean, Toader mean, quadratic mean, complete elliptic integral

## Abstract

In this paper, we present the best possible parameters $\alpha(r)$ and $\beta(r)$ such that the double inequality

$$\begin{aligned} \bigl[\alpha(r)A^{r}(a,b)+ \bigl(1-\alpha(r) \bigr)Q^{r}(a,b) \bigr]^{1/r} < & TD \bigl[A(a,b), Q(a,b) \bigr] \\ < & \bigl[\beta(r)A^{r}(a,b)+ \bigl(1-\beta(r) \bigr)Q^{r}(a,b) \bigr]^{1/r} \end{aligned}$$

holds for all $r\leq 1$ and $a, b>0$ with $a\neq b$, and we provide new bounds for the complete elliptic integral $\mathcal{E}(r)=\int_{0}^{\pi/2}(1-r^{2}\sin^{2}\theta)^{1/2}\,d\theta$ $(r\in (0, \sqrt{2}/2))$ of the second kind, where $TD(a,b)=\frac{2}{\pi}\int_{0}^{\pi/2}\sqrt{a^{2}\cos^{2}\theta+b^{2}\sin^{2}\theta}\,d\theta$, $A(a,b)=(a+b)/2$ and $Q(a,b)=\sqrt{(a^{2}+b^{2})/2}$ are the Toader, arithmetic, and quadratic means of *a* and *b*, respectively.

## Introduction

For $p\in [0, 1]$, $q\in \mathbb{R}$ and $a,b>0$ with $a\neq b$, the *p*th generalized Seiffert mean $S_{p}(a, b)$, *q*th Gini mean $G_{q}(a, b)$, *q*th power mean $M_{q}(a, b)$, *q*th Lehmer mean $L_{q}(a,b)$, harmonic mean $H(a,b)$, geometric mean $G(a,b)$, arithmetic mean $A(a,b)$, quadratic mean $Q(a,b)$, Toader mean $TD(a,b)$ [1], centroidal mean $\overline{C}(a,b)$, contraharmonic mean $C(a,b)$ are, respectively, defined by

1.1 $$\begin{aligned}& S_{p}(a,b)= \textstyle\begin{cases} \displaystyle\frac{p(a-b)}{\arctan [2p(a-b)/(a+b) ]}, &0< p\leq 1,\\ (a+b)/2, &p=0, \end{cases}\displaystyle \\& G_{q}(a,b)= \textstyle\begin{cases} [(a^{q-1}+b^{q-1})/(a+b) ]^{1/(q-2)}, &q\neq 2,\\ (a^{a}b^{b} )^{1/(a+b)}, &q=2, \end{cases}\displaystyle \\& M_{q}(a,b)= \textstyle\begin{cases} [(a^{q}+b^{q})/2 ]^{1/q}, &q\neq 0,\\ \sqrt{ab}, &q=0, \end{cases}\displaystyle \\& L_{q}(a,b)=\frac{a^{q+1}+b^{q+1}}{a^{q}+b^{q}}, \qquad H(a,b)=\frac{2ab}{a+b},\qquad G(a,b)= \sqrt{ab}, \\& A(a,b)=\frac{a+b}{2},\qquad Q(a,b)=\sqrt{\frac{a^{2}+b^{2}}{2}}, \\& TD(a,b)=\frac{2}{\pi} \int_{0}^{{\pi}/{2}}\sqrt{a^{2} \cos^{2}{\theta}+b^{2}\sin^{2}{\theta}}\,d\theta, \\& \overline{C}(a,b)=\frac{2(a^{2}+ab+b^{2})}{3(a+b)}, \qquad C(a,b)=\frac{a^{2}+b^{2}}{a+b}. \end{aligned}$$

It is well known that $S_{p}(a, b)$, $G_{q}(a, b)$, $M_{q}(a, b)$, and $L_{q}(a,b)$ are continuous and strictly increasing with respect to $p\in [0, 1]$ and $q\in \mathbb{R}$ for fixed $a, b>0$ with $a\neq b$, and the inequalities

$$\begin{aligned} H(a,b)&=M_{-1}(a,b)=L_{-1}(a,b)< G(a,b)=M_{0}(a,b)=L_{-1/2}(a,b) \\ & < A(a,b)=M_{1}(a,b)=L_{0}(a,b)< TD(a,b)< \overline{C}(a,b) \\ &< Q(a,b)=M_{2}(a,b)< C(a,b)=L_{1}(a,b) \end{aligned}$$

hold for all $a, b>0$ with $a\neq b$.

The Toader mean $TD(a,b)$ has been well known in the mathematical literature for many years, it satisfies

$$ TD(a,b)=R_{E} \bigl(a^{2}, b^{2} \bigr), $$

where

$$ R_{E}(a,b)=\frac{1}{\pi} \int_{0}^{\infty}\frac{[a(t+b)+b(t+a)]t}{(t+a)^{3/2}(t+b)^{3/2}}\,dt $$

stands for the symmetric complete elliptic integral of the second kind (see [2–4]), therefore it cannot be expressed in terms of the elementary transcendental functions.

Let $r\in (0, 1)$, $\mathcal{K}(r)=\int _{0}^{\pi/2}(1-r^{2}\sin^{2}\theta)^{-1/2}\,d\theta$ and $\mathcal{E}(r)=\int _{0}^{\pi/2}(1-r^{2}\sin^{2}\theta)^{1/2}\,d\theta$ be, respectively, the complete elliptic integrals of the first and second kind. Then $\mathcal{K}(0^{+})=\mathcal{E}(0^{+})=\pi/2$, $\mathcal{K}(r)$, and $\mathcal{E}(r)$ satisfy the derivatives formulas (see [5], Appendix E, p.474-475)

$$\begin{aligned}& \frac{d\mathcal{K}(r)}{dr}=\frac{\mathcal{E}(r)-(1-r^{2})\mathcal{K}(r)}{r(1-r^{2})}, \qquad \frac{d\mathcal{E}(r)}{dr}=\frac{\mathcal{E}(r)-\mathcal{K}(r)}{r}, \\& \frac{d[\mathcal{K}(r)-\mathcal{E}(r)]}{dr}=\frac{r\mathcal{E}(r)}{1-r^{2}}, \end{aligned}$$

the values $\mathcal{K}(\sqrt{2}/2)$ and $\mathcal{E}(\sqrt{2}/2)$ can be expressed as (see [6], Theorem 1.7)

$$ \mathcal{K} \biggl(\frac{\sqrt{2}}{2} \biggr)=\frac{\Gamma^{2} ({1}/{4} )}{4\sqrt{\pi}}=1.854\ldots, \qquad \mathcal{E} \biggl(\frac{\sqrt{2}}{2} \biggr)=\frac{4\Gamma^{2} ({3}/{4} )+\Gamma^{2} ({1}/{4} )}{8\sqrt{\pi}}=1.350 \ldots, $$

where $\Gamma(x)=\int_{0}^{\infty}t^{x-1}e^{-t}\,dt(\operatorname{Re}{x}>0)$ is the Euler gamma function, and the Toader mean $TD(a,b)$ can be rewritten as

1.2 $$ TD(a,b)= \textstyle\begin{cases} {2a}\mathcal{E} (\sqrt{1- ({b}/{a} )^{2}} )/\pi, \quad a\geq b,\\ {2b}\mathcal{E} (\sqrt{1- ({a}/{b} )^{2}} )/\pi, \quad a< b. \end{cases} $$

Recently, the Toader mean $TD(a,b)$ has been the subject of intensive research. Vuorinen [7] conjectured that the inequality

$$ TD(a,b)>M_{3/2}(a,b) $$

holds for all $a, b>0$ with $a\neq b$. This conjecture was proved by Qiu and Shen [8], and Barnard, Pearce and Richards [9], respectively.

Alzer and Qiu [10] presented a best possible upper power mean bound for the Toader mean as follows:

$$ TD(a,b)< M_{\log 2/(\log\pi-\log 2)}(a,b) $$

for all $a, b>0$ with $a\neq b$.

Neuman [2], and Kazi and Neuman [3] proved that the inequalities

$$\begin{aligned}& \frac{(a+b)\sqrt{ab}-ab}{AGM(a,b)}< TD(a,b)< \frac{4(a+b)\sqrt{ab}+(a-b)^{2}}{8AGM(a,b)}, \\& TD(a,b)< \frac{1}{4} \bigl(\sqrt{(2+\sqrt{2})a^{2}+(2- \sqrt{2})b^{2}}+\sqrt{(2+\sqrt{2})b^{2}+(2- \sqrt{2})a^{2}} \bigr) \end{aligned}$$

hold for all $a, b>0$ with $a\neq b$, where $AGM(a,b)$ is the arithmetic-geometric mean of *a* and *b*.

In [11–13], the authors presented the best possible parameters $\lambda_{1}, \mu_{1}\in [0, 1]$ and $\lambda_{2}, \mu_{2}, \lambda_{3}, \mu_{3}\in \mathbb{R}$ such that the double inequalities $S_{\lambda_{1}}(a,b)< TD(a,b)< S_{\mu_{1}}(a,b)$, $G_{\lambda_{2}}(a,b)< TD(a,b)< G_{\mu_{2}}(a,b)$ and $L_{\lambda_{3}}(a,b)< TD(a,b)< L_{\mu_{3}}(a,b)$ hold for all $a, b>0$ with $a\neq b$.

Let $\lambda, \mu, \alpha, \beta\in (1/2, 1)$. Then Chu, Wang and Ma [14], and Hua and Qi [15] proved that the double inequalities

$$\begin{aligned}& C \bigl[\lambda a+(1-\lambda)b, \lambda b+(1-\lambda)a \bigr]< TD(a,b)< C \bigl[ \mu a+(1-\mu)b, \mu b+(1-\mu)a \bigr], \\& \overline{C} \bigl[\alpha a+(1-\alpha)b, \alpha b+(1-\alpha)a \bigr]< TD(a,b)< \overline{C} \bigl[\beta a+(1-\beta)b, \beta b+(1-\beta)a \bigr] \end{aligned}$$

hold for all $a, b>0$ with $a\neq b$ if and only if $\lambda\leq 3/4$, $\mu\geq 1/2+\sqrt{\pi(4-\pi)}/(2\pi)$, $\alpha\leq 1/2+\sqrt{3}/4$ and $\beta\geq 1/2+\sqrt{12/\pi-3}/2$.

In [16–20], the authors proved that the double inequalities

$$\begin{aligned}& \alpha_{1}Q(a,b)+(1-\alpha_{1})A(a,b)< TD(a,b)< \beta_{1}Q(a,b)+(1-\beta_{1})A(a,b), \\& Q^{\alpha_{2}}(a,b)A^{(1-\alpha_{2})}(a,b)< TD(a,b)< Q^{\beta_{2}}(a,b)A^{(1-\beta_{2})}(a,b), \\& \alpha_{3}C(a,b)+(1-\alpha_{3})A(a,b)< TD(a,b)< \beta_{3}C(a,b)+(1-\beta_{3})A(a,b), \\& \frac{\alpha_{4}}{A(a,b)}+\frac{1-\alpha_{4}}{C(a,b)}< \frac{1}{TD(a,b)}< \frac{\beta_{4}}{A(a,b)}+ \frac{1-\beta_{4}}{C(a,b)}, \\& \alpha_{5}C(a,b)+(1-\alpha_{5})H(a,b)< TD(a,b)< \beta_{5}C(a,b)+(1-\beta_{5})H(a,b), \\& \alpha_{6} \bigl[C(a,b)-H(a,b) \bigr]+A(a,b)< TD(a,b)< \beta_{6} \bigl[C(a,b)-H(a,b) \bigr]+A(a,b), \\& \alpha_{7}\overline{C}(a,b)+(1-\alpha_{7})A(a,b)< TD(a,b)< \beta_{7}\overline{C}(a,b)+(1-\beta_{7})A(a,b), \\& \frac{\alpha_{8}}{A(a,b)}+\frac{1-\alpha_{8}}{\overline{C}(a,b)}< \frac{1}{TD(a,b)}< \frac{\beta_{8}}{A(a,b)}+ \frac{1-\beta_{8}}{\overline{C}(a,b)}, \\& \alpha_{9}Q(a,b)+(1-\alpha_{9})H(a,b)< TD(a,b)< \beta_{9}Q(a,b)+(1-\beta_{9})H(a,b), \\& \frac{\alpha_{10}}{H(a,b)}+\frac{1-\alpha_{10}}{Q(a,b)}< \frac{1}{TD(a,b)}< \frac{\beta_{10}}{H(a,b)}+ \frac{1-\beta_{10}}{Q(a,b)} \end{aligned}$$

hold for all $a, b>0$ with $a\neq b$ if and only if $\alpha_{1}\leq 1/2$, $\beta_{1}\geq (4-\pi)/[(\sqrt{2}-1)\pi]$, $\alpha_{2}\leq 1/2$, $\beta_{2}\geq 4-2\log\pi/\log 2$, $\alpha_{3}\leq 1/4$, $\beta_{3}\geq 4/\pi-1$, $\alpha_{4}\leq \pi/2-1$, $\beta_{4}\geq 3/4$, $\alpha_{5}\leq 5/8$, $\beta_{5}\geq 2/\pi$, $\alpha_{6}\leq 1/8$, $\beta_{6}\geq 2/\pi-1/2$, $\alpha_{7}\leq 3/4$, $\beta_{7}\geq 12/\pi-3$, $\alpha_{8}\leq \pi-3$, $\beta_{8}\geq 1/4$, $\alpha_{9}\leq 5/6$, $\beta_{9}\geq 2\sqrt{2}/\pi$, $\alpha_{10}\leq 0$, and $\beta_{10}\geq 1/6$.

The main purpose of this paper is to present the best possible parameters $\alpha(r)$ and $\beta(r)$ such that the double inequality

$$\begin{aligned} \bigl[\alpha(r)A^{r}(a,b)+ \bigl(1-\alpha(r) \bigr)Q^{r}(a,b) \bigr]^{1/r} & < TD \bigl[A(a,b), Q(a,b) \bigr] \\ &< \bigl[\beta(r)A^{r}(a,b)+ \bigl(1-\beta(r) \bigr)Q^{r}(a,b) \bigr]^{1/r} \end{aligned}$$

holds for all $r\leq 1$ and $a, b>0$ with $a\neq b$.

## Lemmas

In order to prove our main result we need two lemmas, which we present in this section.

### Lemma 2.1

*Let* $p\in (0, 1)$, $t\in (0, \sqrt{2}/2)$, $\lambda=(2+\sqrt{2})[1-2\mathcal{E}(\sqrt{2}/2)/\pi]=0.478\ldots$ *and*

2.1 $$ f(t)=\frac{\pi p}{2}\sqrt{1-t^{2}}+\frac{\pi}{2}(1-p)- \mathcal{E}(t). $$

*Then* $f(t)<0$ *for all* $t\in (0, \sqrt{2}/2)$ *if and only if* $p\geq 1/2$ *and* $f(t)>0$ *for all* $t\in (0, \sqrt{2}/2)$ *if and only if* $p\leq \lambda$.

### Proof

It follows from (2.1) that

2.2 $$\begin{aligned}& f \bigl(0^{+} \bigr)=0, \end{aligned}$$

2.3 $$\begin{aligned}& f \biggl(\frac{\sqrt{2}}{2} \biggr)=\frac{\pi}{2} \biggl(1- \frac{\sqrt{2}}{2} \biggr) (\lambda-p), \end{aligned}$$

2.4 $$\begin{aligned}& f^{\prime}(t)=\frac{f_{1}(t)}{t\sqrt{1-t^{2}}}, \end{aligned}$$

where

2.5 $$\begin{aligned}& f_{1}(t)=\sqrt{1-t^{2}} \bigl[\mathcal{K}(t)-\mathcal{E}(t) \bigr]-\frac{\pi p}{2}t^{2},\qquad f_{1} \bigl(0^{+} \bigr)=0, \end{aligned}$$

2.6 $$\begin{aligned}& f_{1} \biggl(\frac{\sqrt{2}}{2} \biggr)=\frac{\sqrt{2}}{2} \biggl[ \mathcal{K} \biggl(\frac{\sqrt{2}}{2} \biggr)-\mathcal{E} \biggl( \frac{\sqrt{2}}{2} \biggr) \biggr] -\frac{\pi p}{4}, \end{aligned}$$

2.7 $$\begin{aligned}& f^{\prime}_{1}(t)=\frac{t[2\mathcal{E}(t)-\mathcal{K}(t)]}{\sqrt{1-t^{2}}}-\pi pt, \end{aligned}$$

2.8 $$\begin{aligned}& f^{\prime}_{1} \bigl(0^{+} \bigr)=0, \end{aligned}$$

2.9 $$\begin{aligned}& f^{\prime}_{1} \biggl(\frac{\sqrt{2}}{2} \biggr)=2\mathcal{E} \biggl(\frac{\sqrt{2}}{2} \biggr)-\mathcal{K} \biggl(\frac{\sqrt{2}}{2} \biggr)- \frac{\sqrt{2}\pi p}{2}, \end{aligned}$$

2.10 $$\begin{aligned}& f^{\prime\prime}_{1}(t)=\frac{ (3-2t^{2} )\mathcal{E}(t)- (2-t^{2} )\mathcal{K}(t)}{ (1-t^{2} )^{3/2}}-\pi p, \end{aligned}$$

2.11 $$\begin{aligned}& f^{\prime\prime}_{1} \bigl(0^{+} \bigr)=\pi \biggl( \frac{1}{2}-p \biggr), \end{aligned}$$

2.12 $$\begin{aligned}& f^{\prime\prime}_{1} \biggl(\frac{\sqrt{2}}{2} \biggr)=\sqrt{2} \biggl[4\mathcal{E} \biggl(\frac{\sqrt{2}}{2} \biggr)-3\mathcal{K} \biggl( \frac{\sqrt{2}}{2} \biggr) \biggr] -\pi p, \end{aligned}$$

2.13 $$\begin{aligned}& f^{\prime\prime\prime}_{1}(t)=-\frac{(1+t^{2})[\mathcal{K}(t)-\mathcal{E}(t)]+t^{2}\mathcal{K}(t)}{t (1-t^{2} )^{5/2}}< 0 \end{aligned}$$

for all $t\in (0, \sqrt{2}/2)$.

It follows from (2.13) that $f_{1}^{\prime\prime}(t)$ is strictly decreasing on $(0, \sqrt{2}/2)$.

We divide the proof into three cases.

*Case 1* $p\geq 1/2$. Then (2.11) leads to

2.14 $$ f^{\prime\prime}_{1} \bigl(0^{+} \bigr)\leq 0. $$

From (2.14) and the monotonicity of $f_{1}^{\prime\prime}(t)$ we clearly see that $f_{1}^{\prime}(t)$ is strictly decreasing on $(0, \sqrt{2}/2)$. Therefore, $f(t)<0$ for all $t\in (0, \sqrt{2}/2)$ follows easily from (2.2), (2.4), (2.5), (2.8), and the monotonicity of $f_{1}^{\prime}(t)$.

*Case 2* $0< p\leq \lambda$. Then from (2.11) and (2.12) together with $4\mathcal{E}(\sqrt{2}/2)-3\mathcal{K}(\sqrt{2}/2)=-0.159\ldots$ we clearly see that

2.15 $$ f^{\prime\prime}_{1} \bigl(0^{+} \bigr)> 0, \qquad f^{\prime\prime}_{1} \biggl(\frac{\sqrt{2}}{2} \biggr)< 0. $$

It follows from (2.15) and the monotonicity of $f_{1}^{\prime\prime}(t)$ that there exists $t_{0}\in (0, \sqrt{2}/2)$ such that $f_{1}^{\prime}(t)$ is strictly increasing on $(0, t_{0}]$ and strictly decreasing on $[t_{0}, \sqrt{2}/2)$.

Let $\lambda^{\ast}=\frac{\sqrt{2}}{\pi} [2\mathcal{E}(\frac{\sqrt{2}}{2})-\mathcal{K}(\frac{\sqrt{2}}{2}) ]=0.381\ldots$ and $\lambda^{\ast\ast}=\frac{2\sqrt{2}}{\pi} [\mathcal{K}(\frac{\sqrt{2}}{2})-\mathcal{E}(\frac{\sqrt{2}}{2}) ]=0.453\ldots$ . We divide the proof into three subcases.

*Subcase 2.1* $0< p\leq\lambda^{\ast}$. Then (2.9) leads to

2.16 $$ f^{\prime}_{1} \biggl(\frac{\sqrt{2}}{2} \biggr)\geq 0. $$

It follows from (2.8) and (2.16) together with the piecewise monotonicity of $f_{1}^{\prime}(t)$ that

2.17 $$ f^{\prime}_{1}(t)>0 $$

for all $t\in (0, \sqrt{2}/2)$.

Therefore, $f(t)>0$ for all $t\in (0, \sqrt{2}/2)$ follows easily from (2.2), (2.4), (2.5), and (2.17).

*Subcase 2.2* $\lambda^{\ast}< p\leq\lambda^{\ast\ast}$. Then (2.6) and (2.9) lead to

2.18 $$\begin{aligned}& f_{1} \biggl(\frac{\sqrt{2}}{2} \biggr)\geq 0, \end{aligned}$$

2.19 $$\begin{aligned}& f^{\prime}_{1} \biggl(\frac{\sqrt{2}}{2} \biggr)< 0. \end{aligned}$$

It follows from (2.8) and (2.19) together with the piecewise monotonicity of $f_{1}^{\prime}(t)$ that there exists $t_{1}\in (0, \sqrt{2}/2)$ such that $f_{1}(t)$ is strictly increasing on $(0, t_{1}]$ and strictly decreasing on $[t_{1}, \sqrt{2}/2)$.

Equation (2.5) and inequality (2.18) together with the piecewise monotonicity of $f_{1}(t)$ lead to the conclusion that

2.20 $$ f_{1}(t)>0 $$

for all $t\in (0, \sqrt{2}/2)$.

Therefore, $f(t)>0$ for all $t\in (0, \sqrt{2}/2)$ follows easily from (2.2) and (2.4) together with (2.20).

*Subcase 2.3* $\lambda^{\ast\ast}< p\leq \lambda$. Then (2.3), (2.6), and (2.9) lead to

2.21 $$\begin{aligned}& f \biggl(\frac{\sqrt{2}}{2} \biggr)\geq 0, \end{aligned}$$

2.22 $$\begin{aligned}& f_{1} \biggl(\frac{\sqrt{2}}{2} \biggr)< 0, \end{aligned}$$

2.23 $$\begin{aligned}& f_{1}^{\prime} \biggl(\frac{\sqrt{2}}{2} \biggr)< 2\mathcal{E} \biggl(\frac{\sqrt{2}}{2} \biggr)-\mathcal{K} \biggl(\frac{\sqrt{2}}{2} \biggr) - \frac{\sqrt{2}\pi}{2}\lambda^{\ast\ast} \\& \phantom{f_{1}^{\prime} \biggl(\frac{\sqrt{2}}{2} \biggr)} < 2\mathcal{E} \biggl(\frac{\sqrt{2}}{2} \biggr)-\mathcal{K} \biggl( \frac{\sqrt{2}}{2} \biggr) -\frac{\sqrt{2}\pi}{2}\lambda^{\ast}=0. \end{aligned}$$

It follows from (2.8) and (2.23) together with the piecewise monotonicity of $f_{1}^{\prime}(t)$ that there exists $t_{2}\in (0, \sqrt{2}/2)$ such that $f_{1}(t)$ is strictly increasing on $(0, t_{2}]$ and strictly decreasing on $[t_{2}, \sqrt{2}/2)$.

From (2.4), (2.5), and (2.22) together with the piecewise monotonicity of $f_{1}(t)$ we clearly see that there exists $t_{3}\in (0, \sqrt{2}/2)$ such that $f(t)$ is strictly increasing on $(0, t_{3}]$ and strictly decreasing on $[t_{3}, \sqrt{2}/2)$.

Therefore, $f(t)>0$ for all $t\in (0, \sqrt{2}/2)$ follows easily from (2.2) and (2.21) together with the piecewise monotonicity of $f(t)$.

*Case 3* $\lambda< p<1/2$. Then (2.3), (2.6), (2.9), (2.11), and (2.12) lead to

2.24 $$\begin{aligned}& f \biggl(\frac{\sqrt{2}}{2} \biggr)< 0, \end{aligned}$$

2.25 $$\begin{aligned}& f_{1} \biggl(\frac{\sqrt{2}}{2} \biggr)< \frac{\sqrt{2}}{2} \biggl[ \mathcal{K} \biggl(\frac{\sqrt{2}}{2} \biggr)-\mathcal{E} \biggl( \frac{\sqrt{2}}{2} \biggr) \biggr] -\frac{\pi \lambda^{\ast\ast}}{4}=0, \end{aligned}$$

2.26 $$\begin{aligned}& f^{\prime}_{1} \biggl(\frac{\sqrt{2}}{2} \biggr)< 2\mathcal{E} \biggl(\frac{\sqrt{2}}{2} \biggr)-\mathcal{K} \biggl(\frac{\sqrt{2}}{2} \biggr)- \frac{\sqrt{2}\pi \lambda^{\ast}}{2}=0, \end{aligned}$$

2.27 $$\begin{aligned}& f^{\prime\prime}_{1} \bigl(0^{+} \bigr)>0, \end{aligned}$$

2.28 $$\begin{aligned}& f^{\prime\prime}_{1} \biggl(\frac{\sqrt{2}}{2} \biggr) < \sqrt{2} \biggl[4\mathcal{E} \biggl(\frac{\sqrt{2}}{2} \biggr)-3\mathcal{K} \biggl( \frac{\sqrt{2}}{2} \biggr) \biggr] -\pi \lambda^{\ast} \\& \phantom{f^{\prime\prime}_{1} \biggl(\frac{\sqrt{2}}{2} \biggr)} =-2\sqrt{2} \biggl[\mathcal{K} \biggl(\frac{\sqrt{2}}{2} \biggr)-\mathcal{E} \biggl(\frac{\sqrt{2}}{2} \biggr) \biggr]< 0. \end{aligned}$$

It follows from (2.27) and (2.28) together with the monotonicity of $f_{1}^{\prime\prime}(t)$ that there exists $t_{4}\in (0, \sqrt{2}/2)$ such that $f_{1}^{\prime}(t)$ is strictly increasing on $(0, t_{4}]$ and strictly decreasing on $[t_{4}, \sqrt{2}/2)$.

Equation (2.8) and inequality (2.26) together with the piecewise monotonicity of $f_{1}^{\prime}(t)$ lead to the conclusion that there exists $t_{5}\in (0, \sqrt{2}/2)$ such that $f_{1}(t)$ is strictly increasing on $(0, t_{5}]$ and strictly decreasing on $[t_{5}, \sqrt{2}/2)$.

From (2.4), (2.5), (2.25), and the piecewise monotonicity of $f_{1}(t)$ we clearly see that there exists $t_{6}\in (0, \sqrt{2}/2)$ such that $f(t)$ is strictly increasing on $(0, t_{6}]$ and strictly decreasing on $[t_{6}, \sqrt{2}/2)$.

Therefore, there exists $t_{7}\in (0, \sqrt{2}/2)$ such that $f(t)>0$ for $t\in (0, t_{7})$ and $f(t)<0$ for $t\in (t_{7}, \sqrt{2}/2)$ follows from (2.2) and (2.24) together with the piecewise monotonicity of $f(t)$. □

### Lemma 2.2

*Let* $r\in \mathbb{R}$, $a, b>0$ *with* $1< b/a<\sqrt{2}$, $c_{0}=2\mathcal{E}(\sqrt{2}/2)/\pi=0.859\ldots$ , $c_{1}=\sqrt{2}/2$, $\lambda(r)$ *and* $U(r; a,b)$ *be defined by*

2.29 $$ \lambda(r)=\frac{1-c^{r}_{0}}{1-c^{r}_{1}} \quad (r\neq 0),\qquad \lambda_{0}= \frac{\log c_{0}}{\log c_{1}} , $$

*and*

2.30 $$ U(r; a,b)= \bigl[\lambda(r)a^{r}+ \bigl(1-\lambda(r) \bigr)b^{r} \bigr]^{1/r} \quad (r\neq 0),\qquad U(0; a,b)=a^{\lambda_{0}}b^{1-\lambda_{0}}, $$

*respectively*. *Then the function* $r\mapsto U(r; a,b)$ *is strictly decreasing on* $(-\infty, \infty)$.

### Proof

Let $x=b/a\in (1, \sqrt{2})$, $r\neq 0$, and

2.31 $$ V(r, x)= \bigl(1-\lambda(r) \bigr)\log x- \bigl(\log \lambda(r) \bigr)^{\prime}. $$

Then from (2.29)-(2.31) one has

2.32 $$\begin{aligned}& \log U(r; a,b)=\log a+\frac{1}{r}\log \bigl(\lambda(r)+ \bigl(1- \lambda(r) \bigr)x^{r} \bigr), \\& \frac{\partial\log U(r; a,b)}{\partial r}=\frac{\lambda^{\prime}(r)(1-x^{r})+(1-\lambda(r))x^{r}\log x}{r (\lambda(r)+(1-\lambda(r))x^{r} )}-\frac{\log (\lambda(r)+(1-\lambda(r))x^{r} )}{r^{2}}, \\& \frac{\partial\log U(r; a,b)}{\partial r}\bigg|_{x=1}=0, \end{aligned}$$

2.33 $$\begin{aligned}& \lambda^{\prime}(r)=\frac{ (c^{r}_{1}-1 )c^{r}_{0}\log c_{0}- (c^{r}_{0}-1 )c^{r}_{1}\log c_{1}}{ (c^{r}_{1}-1 )^{2}}, \\& \bigl(\lambda(r)+ \bigl(1-\lambda(r) \bigr)x^{r} \bigr)|_{x=\sqrt{2}}= \frac{1-c^{r}_{0}}{1-c^{r}_{1}} + \biggl(1-\frac{1-c^{r}_{0}}{1-c^{r}_{1}} \biggr)\frac{1}{c^{r}_{1}}= \frac{c^{r}_{0}}{c^{r}_{1}}, \\& \frac{\lambda^{\prime}(r)(1-x^{r})+(1-\lambda(r))x^{r}\log x}{r (\lambda(r)+(1-\lambda(r))x^{r} )}\bigg|_{x=\sqrt{2}}=\frac{1}{r}\log\frac{c_{0}}{c_{1}}, \\& \frac{\partial\log U(r; a, b)}{\partial r}\bigg|_{x=\sqrt{2}}=0, \end{aligned}$$

2.34 $$\begin{aligned}& \frac{\partial^{2}\log U(r; a, b)}{\partial x\,\partial r}=\frac{\lambda(r)x^{r-1}}{ (\lambda(r)+(1-\lambda(r))x^{r} )^{2}}V(r, x), \end{aligned}$$

2.35 $$\begin{aligned}& V(r, 1)=\frac{\log\frac{1}{c_{1}}}{ (\frac{1}{c_{1}} )^{r}-1}-\frac{\log\frac{1}{c_{0}}}{ (\frac{1}{c_{0}} )^{r}-1}< 0, \end{aligned}$$

2.36 $$\begin{aligned}& V(r, \sqrt{2})=c^{r}_{0} \biggl(\frac{\log c_{1}}{c^{r}_{1}-1}- \frac{\log c_{0}}{c^{r}_{0}-1} \biggr)>0, \end{aligned}$$

where inequalities (2.35) and (2.36) hold due to $c_{0}>c_{1}$ and the function $t\mapsto \log t/(t^{r}-1)$ is strictly decreasing on $(0, \infty)$.

Note that $\lambda(r)\in (0, 1)$ and the function $x\rightarrow V(r, x)$ is strictly increasing on $(1, \sqrt{2})$. Then (2.34)-(2.36) lead to the conclusion that there exists $x_{0}\in (1, \sqrt{2})$ such that the function $x\mapsto \partial \log U(r; a, b)/\partial r$ is strictly decreasing on $(1, x_{0})$ and strictly increasing on $(x_{0}, \sqrt{2})$.

It follows from (2.32) and (2.33) together with the piecewise monotonicity of the function $x\mapsto \partial \log U(r; a, b)/\partial r$ on the interval $(1, \sqrt{2})$ that

2.37 $$ \frac{\partial\log U(r; a, b)}{\partial r}< 0 $$

for all $a, b>0$ with $1< b/a<\sqrt{2}$.

Therefore, Lemma 2.2 follows from (2.37). □

## Main result

### Theorem 3.1

*Let* $c_{0}=2\mathcal{E}(\sqrt{2}/2)/\pi=0.859\ldots$ , $c_{1}=\sqrt{2}/2$ *and* $\lambda(r)$ *be defined by* (2.29). *Then the double inequality*

$$\begin{aligned} \bigl[\alpha(r)A^{r}(a,b)+ \bigl(1-\alpha(r) \bigr)Q^{r}(a,b) \bigr]^{1/r}&< TD \bigl[A(a,b), Q(a,b) \bigr] \\ &< \bigl[\beta(r)A^{r}(a,b)+ \bigl(1-\beta(r) \bigr)Q^{r}(a,b) \bigr]^{1/r} \end{aligned}$$

*holds for all* $r\leq 1$ *and* $a, b>0$ *with* $a\neq b$ *if and only if* $\alpha(r)\geq 1/2$ *and* $\beta(r)\leq \lambda(r)$, *where* $r=0$ *is the limit value of* $r\rightarrow 0$.

### Proof

We first prove that Theorem 3.1 holds for $r=1$.

Since $A(a,b)< TD[A(a,b), Q(a,b)]< Q(a,b)$ for all $a, b>0$ with $a\neq b$, and $A(a,b)$, $TD(a,b)$ and $Q(a,b)$ are symmetric and homogeneous of degree 1, without loss of generality, we assume that $\alpha(1)$, $\beta(1)\in (0, 1)$ and $a>b$. Let $t=(a-b)/\sqrt{2(a^{2}+b^{2})}\in (0, \sqrt{2}/2)$ and $p\in (0, 1)$. Then (1.1) and (1.2) lead to

3.1 $$\begin{aligned}& A(a,b)=Q(a,b)\sqrt{1-t^{2}}, \qquad TD \bigl[A(a,b), Q(a,b) \bigr]= \frac{2}{\pi}Q(a,b)\mathcal{E}(t), \\ & \\ & pA(a,b)+(1-p)Q(a,b)-TD \bigl[A(a,b), Q(a,b) \bigr]=\frac{2}{\pi}Q(a,b)f(t), \end{aligned}$$

where $f(t)$ is defined as in Lemma 2.1.

Therefore, Theorem 3.1 for $r=1$ follows easily from Lemma 2.1 and (3.1).

Next, let $r<1$ and $a, b>0$ with $a\neq b$, then it follows from Theorem 3.1 for $r=1$ that

3.2 $$ \frac{A(a,b)+Q(a,b)}{2}< TD \bigl[A(a,b), Q(a,b) \bigr]< \lambda(1)A(a,b)+ \bigl(1- \lambda(1) \bigr)Q(a,b). $$

Note that

3.3 $$\begin{aligned}& 1< \frac{Q(a,b)}{A(a,b)}< \sqrt{2}, \end{aligned}$$

3.4 $$\begin{aligned}& \frac{TD[A(a,b), Q(a,b)]}{ [\frac{A^{r}(a,b)+Q^{r}(a,b)}{2} ]^{1/r}}=\frac{2^{1+1/r}}{\pi}\frac{\mathcal{E}(t)}{ [1+(1-t^{2})^{r/2} ]^{1/r}}, \end{aligned}$$

3.5 $$\begin{aligned}& \frac{TD[A(a,b), Q(a,b)]}{ [\lambda(r)A^{r}(a,b)+(1-\lambda(r))Q^{r}(a,b) ]^{1/r}} =\frac{2}{\pi}\frac{\mathcal{E}(t)}{ [\lambda(r)(1-t^{2})^{r/2}+1-\lambda(r) ]^{1/r}}, \end{aligned}$$

3.6 $$\begin{aligned}& \lim_{t\rightarrow 0^{+}}\frac{2^{1+1/r}}{\pi}\frac{\mathcal{E}(t)}{ [1+(1-t^{2})^{r/2} ]^{1/r}} =\lim _{t\rightarrow \sqrt{2}/2}\frac{2}{\pi}\frac{\mathcal{E}(t)}{ [\lambda(r)(1-t^{2})^{r/2}+1-\lambda(r) ]^{1/r}}=1. \end{aligned}$$

Therefore, Theorem 3.1 for $r<1$ follows from (3.2)-(3.6) and Lemma 2.2 together with the monotonicity of the function $r\mapsto [(a^{r}+b^{r})/2]^{1/r}$. □

Let $r=1$. Then Theorems 3.1 leads to Corollary 3.2 immediately.

### Corollary 3.2

*Let* $\lambda=(2+\sqrt{2})[1-2\mathcal{E}(\sqrt{2}/2)/\pi]$. *Then the double inequality*

$$ \frac{\pi}{4}\sqrt{1-t^{2}}+\frac{\pi}{4}< \mathcal{E}(t)< \frac{\pi}{2}\lambda\sqrt{1-t^{2}}+\frac{\pi}{2}(1-\lambda) $$

*holds for all* $t\in (0, \sqrt{2}/2)$.
